# The role of clopidogrel resistance-related genetic and epigenetic factors in major adverse cardiovascular events among patients with acute coronary syndrome after percutaneous coronary intervention

**DOI:** 10.3389/fcvm.2022.1027892

**Published:** 2023-02-08

**Authors:** Astuti Giantini, Ina S. Timan, Rahajuningsih Dharma, Renan Sukmawan, Rianto Setiabudy, Idrus Alwi, Alida R. Harahap, Erlin Listiyaningsih, Lia G. Partakusuma, Arif R. Tansir, Windy Sahar, Rakhmad Hidayat

**Affiliations:** ^1^Clinical Pathology Department, Faculty of Medicine, Universitas Indonesia, Dr. Cipto Mangunkusumo National Public Hospital, Central Jakarta, Indonesia; ^2^Universitas Indonesia Hospital, Universitas Indonesia, Depok, Indonesia; ^3^Cardiology and Vascular Medicine Department, Faculty of Medicine, Universitas Indonesia, National Cardiovascular Center Harapan Kita, West Jakarta, Indonesia; ^4^Pharmacology and Therapeutics Department, Faculty of Medicine, Universitas Indonesia, Dr. Cipto Mangunkusumo National Public Hospital, Central Jakarta, Indonesia; ^5^Internal Medicine Department, Faculty of Medicine, Universitas Indonesia, Dr. Cipto Mangunkusumo National Public Hospital, Central Jakarta, Indonesia; ^6^National Cardiovascular Center Harapan Kita, West Jakarta, Indonesia; ^7^Faculty of Medicine, Universitas Indonesia, Central Jakarta, Indonesia; ^8^Neurology Department, Faculty of Medicine, Universitas Indonesia, Dr. Cipto Mangunkusumo National Public Hospital, Central Jakarta, Indonesia

**Keywords:** acute coronary syndrome, clopidogrel resistance, epigenetic factor, genetic factor, major adverse cardiovascular events

## Abstract

Despite patients with acute coronary syndrome (ACS) undergoing percutaneous coronary intervention (PCI) and receiving clopidogrel therapy, some patients still experience major adverse cardiovascular events (MACEs). Clopidogrel resistance, which may be regulated by genetic and epigenetic factors, may play a role in MACEs. This study aimed to determine the association between genetic (*CYP2C19* and *P2Y12* polymorphisms) and epigenetic (DNA methylation of *CYP2C19* and *P2Y12* and miRNA-26a expression) factors and their effects on MACEs among post-PCI patients. Post-PCI patients who received a standard dosage of clopidogrel at Harapan Kita Hospital between September 2018 and June 2020 were included in this study. MACEs were observed in patients within 1 year after PCI. Platelet aggregation was assessed using light transmission aggregometry (LTA). DNA methylation of *CYP2C19* and *P2Y12* was assessed using the bisulfite conversion method. *CYP2C19* and *P2Y12* polymorphisms and miRNA-26a expression were evaluated using quantitative real-time polymerase chain reaction (qRT-PCR). Among a total of 201 subjects, 49.8% were clopidogrel-resistant, and 14.9% experienced MACEs within 1 year after PCI (death was 7.5%). Hypomethylation of *CYP2C19* (*p* = 0.037) and miRNA-26a upregulation (*p* = 0.020) were associated with clopidogrel resistance. CYP2C19^*^2/^*^3 polymorphisms (*p* = 0.047) were associated with MACEs in 1 year. This study demonstrated that hypomethylation of *CYP2C19* and miRNA-26a upregulation increased the risk of clopidogrel resistance in post-PCI patients, but there was no correlation between clopidogrel resistance and MACEs. However, CYP2C19^*^2/^*^3 polymorphisms were the factors that predicted MACEs within 1 year.

## 1. Introduction

Coronary heart diseases (CHDs), which encompass acute coronary syndrome (ACS) and myocardial infarction (MI), continue to be a major global health problem and the leading cause of death worldwide, accounting for 16% of the 55.4 million total deaths worldwide in 2019 (1). Percutaneous coronary intervention (PCI) is a non-surgical revascularization method that is commonly performed to restore coronary blood flow (2). Patients undergoing PCI are still at risk of developing major adverse cardiac events (MACEs), such as recurrent angina pectoris, recurrent acute myocardial infarction (AMI), stroke, and death (3–5). A combination of aspirin and clopidogrel (dual antiplatelet therapy) has become a standard pharmacotherapeutic modality to prevent the onset and recurrence of ischemic events, thereby reducing MACEs (2, 6). Inadequate clopidogrel response leads to decreased inhibition of platelets, a condition known as clopidogrel resistance, which is quite common (7). Studies in Asia showed that the prevalence of clopidogrel resistance was as high as 20–65% according to the platelet aggregation test (8). Some studies have demonstrated the association between clopidogrel response and ischemic events (9, 10). The clopidogrel resistance in-stent thrombosis (CREST) study has shown the association of clopidogrel resistance with in-stent thrombosis (9). In a case report, antiplatelet agent substitution guided by resistance information is shown to reduce the incidence of in-stent restenosis (10).

Clopidogrel is a prodrug activated by liver cytochrome P450, particularly *CYP2C19*. Clopidogrel inhibits adenosine diphosphate (ADP) *P2Y12* receptors on platelets (6). Clopidogrel response is regulated by several factors, such as drug interactions, compliance, comorbidities, and genetic and epigenetic factors (11–19). *CYP2C19* polymorphisms marked by CYP2C19^*^2 and CYP2C19^*^3 loss-of-function alleles lead to decreased enzymatic activity related to the biotransformation of clopidogrel (11, 12). The *P2Y12* polymorphism is also associated with an increased risk of clopidogrel resistance (13). Nowadays, epigenetic factors are known to be involved in the regulation of drug responses, degenerative disorders, and cancers (14). Two main mechanisms of epigenetic factors are deoxyribonucleic acid (DNA) methylation and micro-ribonucleic acid (miRNA). DNA methylation is a crucial marker in the regulation of gene expression. Because *CYP2C19* is the predominant isoenzyme in the biotransformation of clopidogrel and the *P2Y12* receptor is the target of clopidogrel, DNA methylation of *CYP2C19* and *P2Y12* may affect the risk of clopidogrel resistance (15). miRNA-26a expression regulates vasodilator-stimulated phosphoprotein (VASP) expression that controls actin, which plays a role in the mechanism of platelet aggregation (16). However, there is still limited evidence on the association between genetic and epigenetic factors and clopidogrel resistance. In addition, there are still limited studies that directly show the relationship between genetic and epigenetic factors with clinical outcomes. Because gene polymorphisms are hereditary and irreversible, their profile can be identified before the administration of antiplatelets (17). Therefore, the objective of this study was to determine the association between genetic and epigenetic factors, such as *CYP2C19* and *P2Y12* polymorphisms, DNA methylation of *CYP2C19* and *P2Y12*, and miRNA-26a expression with clopidogrel resistance, and MACEs among post-PCI patients in a 1-year observation.

## 2. Materials and methods

### 2.1. Study population

This study was conducted between September 2018 and June 2020 at the National Cardiovascular Center Harapan Kita. Subjects were post-PCI patients who had ACS and received clopidogrel therapy, with a minimum sample size of 200 patients determined by the rule-of-thumb equation for 20 independent variables. Included designs were cross-sectional and prospective cohort for the clopidogrel resistance study and the MACE study, respectively. Inclusion criteria included (1) post-PCI patients who had ACS and received clopidogrel 75 mg daily at least 6 h after the loading dose (during hospitalization and later); (2) those taking clopidogrel regularly; and (3) those who had signed informed consent. Exclusion criteria included (1) thrombocytopenia; (2) thrombocytosis; (3) hemolytic, lipemic, or icteric blood samples; and (4) the presence of hemorrhagic manifestations. This study was approved by the Ethics Committee of the National Cardiovascular Center, Harapan Kita Hospital. Subjects were prospectively observed for 1 year through monthly telephonic interviews. Subjects were required to report to the National Cardiovascular Center, Harapan Kita, on a monthly basis after PCI for anamnesis, physical examination, and continuation of clopidogrel medication. Demographic data, cardiovascular risk factors, and laboratory results were obtained. Angina pain, recurrent acute myocardial infarction (AMI), stroke, or death within 1 year were recorded as MACEs. A subject who could not be reached after 1 year of observation was considered a dropout.

### 2.2. Blood sample and platelet aggregation test

Approximately 15 ml of venous blood was drawn from subjects. Then, 9 ml of blood was divided into three tubes with sodium citrate for a platelet aggregation test to determine clopidogrel resistance. Blood samples were centrifuged for the platelet aggregation test to obtain platelet-rich plasma (PRP). The agonist ADP 20 μM was added to PRP. Light transmission aggregometry (LTA) using the Agram aggregometer method was used for the platelet aggregation test, where platelet aggregation greater than 59% was defined as clopidogrel-resistant (20). A within-run accuracy test was performed on the platelet aggregation test. *CYP2C19* and *P2Y12* gene polymorphisms and miRNA-26a expression were evaluated using quantitative real-time polymerase chain reaction (qRT-PCR), and DNA methylation of *CYP2C19* and *P2Y12* genes was assessed using the bisulfite conversion method. The remaining 6 ml of blood were split into two tubes with ethylenediaminetetraacetic acid (EDTA) for the analysis of *CYP2C19* and *P2Y12* polymorphisms, DNA methylation of *CYP2C19* and *P2Y12*, and miRNA-26a expression.

### 2.3. Polymorphism assay

Single-nucleotide polymorphism (SNP) of CYP2C19 was identified as CYP2C19^*^2 (G681A; rs4244285) and CYP2C19^*^3 (G636A; rs4986893), while SNP of *P2Y12* was identified as the A57T polymorphism (rs3679479). First, peripheral blood mononuclear cells (PBMCs) were isolated from the blood sample. DNA was obtained by extracting PBMC using the QIAamp DNA mini kit. *CYP2C19* polymorphism assay was performed using the Taqman assay kit (ThermoFisher Scientific). The method used was qRT-PCR. If polymorphisms were identified, a mutant carrier of the *CYP2C19* polymorphism was determined.

### 2.4. DNA methylation assay

DNA methylation of *CYP2C19* was found in the gene body, while DNA methylation of *P2Y12* was found in the promoter. 5′-cytosine-phosphate-guanine-3′ (CpG) islands in the *CYP2C19* gene body and its primer design were identified using Methyl Primer Express and Refseq CYP2C19 software; three CpG islands were found. The P2Y12 primer was designed according to Li et al. (19) (Table 1). First, bisulfite conversion of DNA using EpiTech Bisulfite kits resulted in the deamination of unmethylated cytosines to uracils without changing methylated cytosines. Following that, qRT-PCR and high-resolution melting (HRM) analyses were performed. Then, the percentage of DNA methylation was obtained. Hypermethylation and hypomethylation of *CYP2C19* were defined as methylation levels greater and less than 50%, respectively.

**Table 1:** Primer design.

**Gene**	**Group**	**Primer Sequence**
*CYP2C19*	Forward	5′ TTAGTGAGATTTCGTGGGC 3′
	Reverse	5′ ATACGTACACCCTACGAAAACC 3′
*P2Y12*	Forward	5′-TATTTGGAATTTATTTGGATGTGTG-3′
	Reverse	5′-AATTCAAAACCAACCTAACCAAAAT-3′

5′-cytosisne-phosphate-guanine-3′ (CpG) islands in the *CYP2C19* gene body and its primer design were determined using Methyl Primer Express and Refseq CYP2C19 software; three CpG islands were found, and the *P2Y12* primer was designed according to Li et al. (19).

### 2.5. miRNA-26a expression assay

The first step in the miRNA-26a expression assay was miRNA isolation using the miRNeasy Mini kit Qiagen. Isolated miRNA was converted into complementary DNA (cDNA) using TaqMan miRNA reverse transcription. Then, cDNA qRT-PCR was performed. The analysis of miRNA-26a expression was determined by comparing its ΔΔCT and positive control. miRNA-26a upregulation and downregulation were defined as high and low positive controls, respectively.

### 2.6. Statistical analysis

IBM SPSS Statistics 22.0 was used for statistical analysis. The chi-square or Fisher's exact test was used in a bivariate analysis between several factors and clopidogrel resistance. Logistic regression was used in a multivariate analysis of factors that contributed to clopidogrel resistance. A Cox regression was used in bivariate and multivariate analyses to find the association between several factors and MACEs in 1 year. Statistical significance was defined as a *p-*value of <0.05.

## 3. Results

### 3.1. Characteristics of subjects

Between September 2018 and June 2020, a total of 201 patients were included. Clopidogrel resistance was found in 49.8% of patients. Baseline characteristics of the subjects are presented in Table 2 (overall and based on genetic factors), and laboratory parameters are presented in Table 3. Based on genetic and epigenetic factors, 45.8% were mutant carriers of CYP2C19^*^2/^*^3, 36.8% were mutant carriers of the *P2Y12* polymorphism, 80.1% had hypomethylation of *CYP2C19*, 10% had hypomethylation of *P2Y12*, and 66.2% had miRNA-26a upregulation. A within-run accuracy test of platelet aggregation found that the coefficient of variance (CV) in the clopidogrel resistance group and the non-clopidogrel resistance group was 2.02 and 7.45%, respectively. After 1 year of observation, 30 subjects (14.9%) developed MACEs; with deaths (7.5%) being the most frequent MACEs.

**Table 2:** Subjects' characteristics.

**Variables**	***n*** **(%)**				
	**Overall**	**CYP2C19 mutant carrier**	**CYP2C19 wildtype**	**P2Y12 mutant carrier**	**P2Y12 wildtype**
**Demographic factors (*****n** =* **201)** **Sex**					
Male	186 (92.5)	86 (46.2)	100 (53.8)	70 (37.6)	116 (62.4)
Female	15 (7.5)	6 (40)	9 (60)	4 (26.7)	11 (73.3)
**Age, y**					
**Age group**					
≥60 y	54 (26.9)	28 (51.9)	26 (48.1)	18 (33.3)	36 (66.7)
<60 y	147 (73.1)	64 (43.5)	83 (56.5)	56 (38.1)	91 (61.9)
**Nutritional status**					
Obese	108 (53.7)	51 (47.2)	57 (52.8)	41 (38)	67 (62)
Overweight	43 (21.4)	18 (41.9)	25 (58.1)	13 (30.2)	30 (69.8)
Normoweight	50 (24.9)	23 (46)	27 (54)	20 (40)	60 (30)
**Cardiovascular risk factors (*****n** =* **201)**					
Hypertension	187 (93,0)	84 (44.9)	103 (55.1)	67 (35.8)	120 (64.2)
Diabetes mellitus	79 (39,3)	32 (40.5)	47 (59.5)	34 (43)	45 (57)
Family history	25 (12,4)	11 (44)	14 (56)	7 (28)	18 (72)
Smoking	136 (67,7)	58 (42.6)	78 (57.4)	52 (38.2)	84 (61.8)
Dyslipidemia	48 (23,9)	24 (50)	24 (50)	23 (47.9)	25 (52.1)
**MACEs (*****n** =* **201)**	**30 (14.9)**				
Angina pectoris	7 (3.5)				
Recurrent AMI	7 (3.5)				
Stroke	1 (0.5)				
Death	15 (7.5)				

AMI, acute myocardial infarction; MACEs, major adverse cardiovascular events.

**Table 3:** Laboratory parameters.

**Variables**	***n* (%)**
**Laboratory value (*****n** =* **201)** **Total cholesterol**		
High	33 (16.4)
Normal	168 (83.6)
**HDL**	
Low	183 (91.0)
Normal	18 (9.0)
**LDL**	
High	132 (65.7)
Normal	69 (34.3)
**Triglyceride**	
High	50 (24.9)
Normal	151 (75.1)
**Hemoglobin**	
Low	31 (15.4)
Normal	170 (84.6)
**Leukocyte**	
High	171 (85.1)
Normal	30 (14.9)
**eGFR**	
Low	37 (18.4)
Normal	164 (81.6)
**Genetic factors (*****n** =* **201)** CYP2C19^*^2 and CYP2C19^*^3		
Mutant carrier	92 (45.8)
Homozygous wildtype	109 (54.2)
**Epigenetic factors (*****n** =* **201)** **CYP2C19 DNA methylation**		
Hypomethylation	161 (80.1)
Hypermethylation	40 (19.9)
**P2Y12 DNA methylation**	
Hypomethylation	20 (10.0)
Hypermethylation	181 (90.0)
**miRNA-26a expression**	
Upregulated	133 (66.2)
Downregulated	68 (33.8)
**Platelet aggregation test (clopidogrel resistance) (*****n** =* **201)**	
≥59% (resistance)	100 (49.8)
<59% (non-resistance)	101 (50.2)

Based on CYP2C19 polymorphism, 45.8% were mutant carriers of CYP2C19^*^2/^*^3 and clopidogrel resistance proportion was quite high (49.8%). eGFR, estimated glomerular filtration rate; HDL, high-density lipoprotein; LDL, low-density lipoprotein.

### 3.2. Association between genetic and epigenetic factors and clopidogrel resistance

As presented in Table 4, DNA methylation of CYPC19 and miRNA-26a expression were associated with clopidogrel resistance. Hypomethylation of *CYP2C19* [odds ratio (OR) = 2.13, 95% confidence interval (CI) = 1.04–4.37, *p*-value = 0.037] and miRNA-26a upregulation (OR = 2.03, 95%CI = 1.12–3.68, *p*-value = 0.020) were associated with an increase in the risk of clopidogrel resistance. However, there was no association between other genetic and epigenetic factors and clopidogrel resistance. Logistic regression analysis was performed to identify clinical factors, laboratory parameters, and genetic and epigenetic factors. From the logistic regression analysis, DNA methylation of *CYP2C19* and miRNA-26a expression were found to be independent factors of clopidogrel resistance

**Table 4:** The association between several factors and clopidogrel resistance.

**Variables**	**LTA ≥59% (Clopidogrel resistance) (*n =* 100)**	**LTA <59% (Clopidogrel non- resistance) (*n =* 101)**	**OR (95% CI)**	***P*-value**	**Adjusted OR (95% CI)**	***P*-value**
**Demographic factors**						
Male, *n* (%)	91 (49.9)	95 (51.1)	1.57 (0.54–4.58)	0.409		
Age ≥ 60 y, *n* (%)	30 (55.6)	24 (44.4)	1.38 (0.74–2.57)	0.319		
Obese, *n* (%)	58 (53.7)	50 (46.3)	1.12 (0.80–1.57)	0.553		
**Cardiovascular risk factors**						
Hypertension, *n* (%)	93 (49.7)	94 (50.3)	0.99 (0.33–2.93)	0.985		
Diabetes mellitus, *n* (%)	41 (51.9)	38 (48.1)	1.15 (0.65–2.03)	0.624		
Family history, *n* (%)	14 (56.0)	11 (44.0)	1.33 (0.57-3.10)	0.504		
Smoking, *n* (%)	57 (41.9)	79 (58.1)	0.37 (0.20–0.68)	0.001	0.36 (0.19–0.67)	0.001
Dyslipidemia, *n* (%)	28 (58.3)	20 (41.7)	1.58 (0.82–3.03)	0.173		
**Laboratory parameters**						
High total cholesterol, *n* (%)	18 (54.5)	15 (45.5)	1.26 (0.60–2.66)	0.547		
Low HDL, *n* (%)	89 (48.6)	94 (51.4)	0.60 (0.22–1.62)	0.312		
High LDL, *n* (%)	64 (48.5)	68 (51.5)	0.86 (0.48–1.55)	0.619		
High triglyceride, *n* (%)	28 (56.0)	22 (44.0)	1.40 (0.73–2.66)	0.308		
Low hemoglobin, *n* (%)	19 (61.3)	12 (38.7)	1.74 (0.80–3.81)	0.162		
High leukocyte, *n* (%)	87 (50.9)	84 (49.1)	1.35 (0.62–2.96)	0.446		
Low eGFR, *n* (%)	23 (62.2)	14 (37.8)	1.86 (0.89–3.86)	0.095		
**CYP2C19 polymorphism**, ***n*** **(%)**						
Hetero/homozygous ^*^2 and/or ^*^3	51 (55.4)	41 (44.6)	1.52 (0.87–2.66)	0.139		
Homozygous wildtype	49 (45,0)	60 (55,0)				
**CYP2C19 DNA methylation**, ***n*** **(%)**						
Hypomethylation	86 (46.6)	75 (53.4)	2.13 (1.04–4.37)	0.037	2.14 (1.01–4.55)	0.048
Hypermethylation	14 (35.0)	26 (65.0)				
**P2Y12 DNA methylation**, ***n*** **(%)**						
Hypomethylation	12 (60.0)	8 (40.0)	1.59 (0.62–4.06)	0.334		
Hypermethylation	88 (48.6)	93 (51.4)				
**miRNA-26a expression**, ***n*** **(%)**						
Upregulated	74 (55.6)	59 (44.4)	2.03 (1.12–3.68)	0.020	2.10 (1.13–3.92)	0.020
Upregulated	26 (38.2)	42 (61.8)				

eGFR, estimated glomerular filtration rate; HDL, high-density lipoprotein; LDL, low-density lipoprotein; MACEs, major adverse cardiovascular events; OR, odds ratio.

### 3.3. Association between genetic and epigenetic factors and MACEs

**Figure 1:**
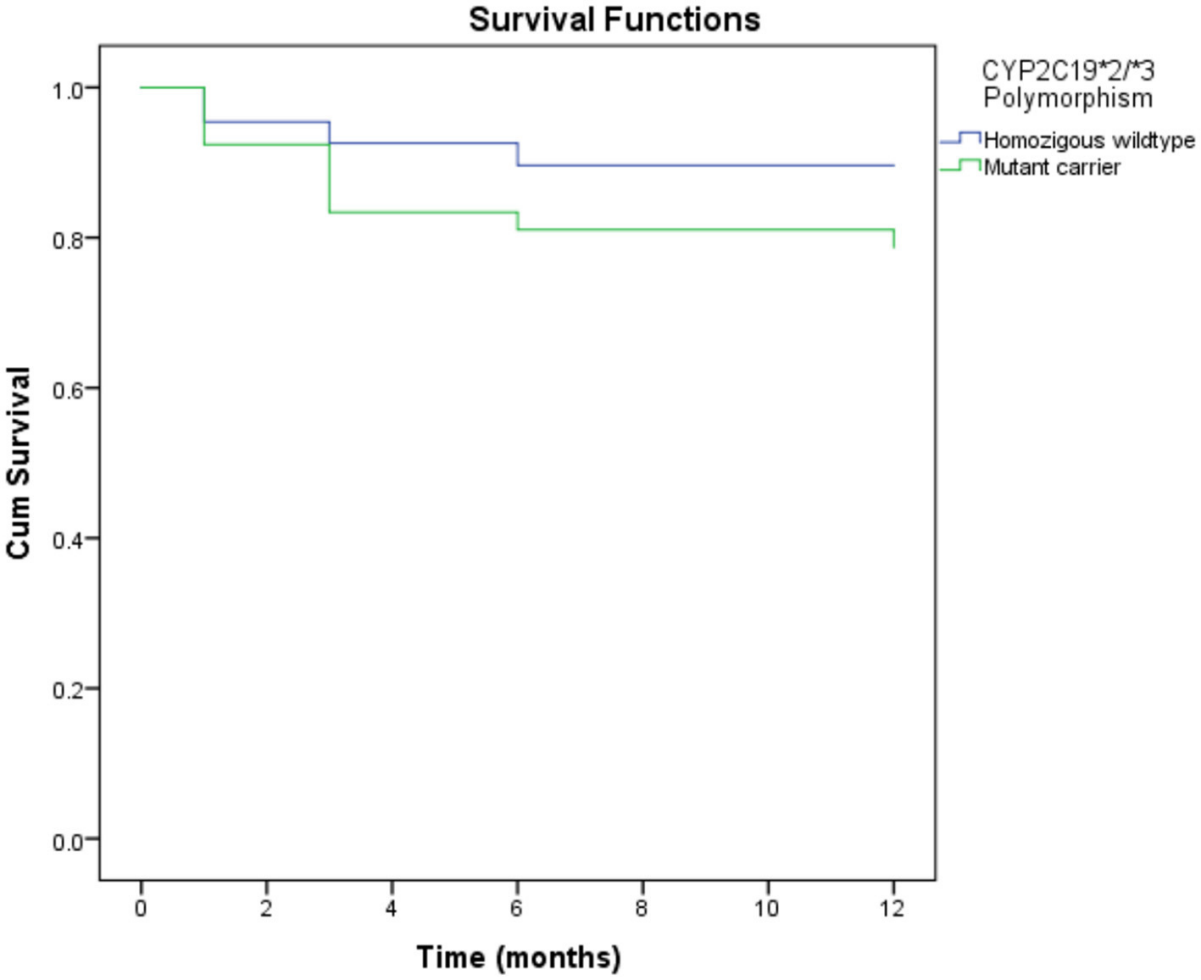
Kaplan-Meier curves of MACE in 1 year according to CYP2C19*2 and CYP2C19*3 polymorphisms. Mutant carrier of CYP2C19*2/*3 polymorphism resulted in developing MACE faster than wild type in 1 year of observation (HR = 2.12, 95%CI = 1.01–4.46, *P*-value = 0.047). MACE, major adverse cardiovascular events.

As shown in Table 5, the *CYP2C19* polymorphism was associated with MACEs in 1 year. The mutant carrier of CYP2C19^*^2/^*^3 [hazard ratio (HR) = 2.12, 95%CI = 1.01–4.46, *p*-value = 0.047] was associated with an increase in MACEs in 1 year. However, there was no association between other genetic and epigenetic factors and MACEs. Gender and age were associated with MACEs in 1 year. Instead of the male gender, the female gender was associated with an increased risk of MACEs (HR = 2.73, 95%CI = 1.05–7.14, *p*-value = 0.040). Age over 60 was also associated with an increased risk of MACEs (HR = 2.17, 95%CI = 1.06–4.48, *p*-value = 0.035). The laboratory parameter associated with MACEs was the estimated glomerular filtration rate (eGFR). A low eGFR was associated with an increased risk of MACEs (HR = 3.29, 95%CI = 1.59–6.84, *p*-value = 0.001). However, gender and age were not the factors that predicted MACEs in multivariate analysis. Although leukocytes were not associated with MACEs in bivariate analysis, the predictors of MACEs based on multivariate analysis were highly leukocytes, eGFR, and the *CYP2C19* polymorphism. Figure 1 shows a mutant carrier of CYP2C19^*^2/^*^3 that develops MACEs faster than the wildtype in a 1-year observation.

**Table 5:** The association between several factors and MACEs in 1 year.

**Variables**	**MACEs** **(*n =* 30)**	**Non-MACEs (*n =* 171)**	**HR (95% CI)**	***P*-value**	**Adjusted HR (95% CI)**	***P*-value**
**Demographic Factors**						
Female, *n* (%)	5 (33.3)	10 (66.7)	2.73 (1.05–7.14)	0.040		
Age ≥ 60 y, *n* (%)	13 (24.1)	41 (75.9)	2.17 (1.06–4.48)	0.035		
Obese, *n* (%)	17 (15.7)	91 (84.3)	0.92 (0.60–1.40)	0.692		
**Cardiovascular risk factors**						
Hypertension, *n* (%)	29 (15.5)	158 (84.5)	2.19 (0.30–16.09)	0.440		
Diabetes mellitus, *n* (%)	12 (15.2)	67 (84.8)	1.05 (0.51–2.18)	0.895		
Smoking, *n* (%)	17 (12.5)	119 (87.5)	0.61 (0.30–1.25)	0.176		
Family history, *n* (%)	4 (16.0)	21 (84.0)	1.09 (0.38–23.11)	0.878		
Dyslipidemia, *n* (%)	5 (10.4)	43 (89.6)	0.62 (0.24–1.62)	0.331		
**Laboratory parameters**						
High total cholesterol, *n* (%)	4 (12.1)	29 (87.9)	0.78 (0.27–2.22)	0.637		
Low HDL, *n* (%)	29 (15.8)	154 (84.2)	2.91 (0.40–21.38)	0.293		
High LDL, *n* (%)	20 (15.2)	112 (84.8)	1.07 (0.50–2.28)	0.871		
High triglyceride, *n* (%)	10 (20.0)	40 (80.0)	1.57 (0.74–3.36)	0.244		
Low hemoglobin, *n* (%)	5 (16.1)	26 (83.9)	1.13 (0.43–2.95)	0.803		
High leukocyte, *n* (%)	29 (17.0)	142 (83.0)	5.37 (0.73–39.43)	0.098	7.59 (1.03–56.10)	0.047
Low eGFR, *n* (%)	12 (32.4)	25 (67.6)	3.29 (1.59–6.84)	0.001	4.05 (1.94–8.46)	0.000
Platelet aggregation test (clopidogrel resistance), *n* (%)	19 (19.0)	81 (81.0)	1.80 (0.86–3.78)	0.121		
CYP2C19^*^2 and CYP2C19^*^3, ***n*** **(%)**						
Mutant carrier	19 (20.7)	73 (79.3)	2.12 (1.01–4.46)	0.047	2.60 (1.23–5.49)	0.012
Homozygous wildtype	11 (10.1)	98 (89.9)				

eGFR, estimated glomerular filtration rate; HDL, high-density lipoprotein; HR, hazard ratio; LDL, low-density lipoprotein; MACEs, major adverse cardiovascular events.

## 4. Discussion

### 4.1. Association between several factors and clopidogrel resistance

In this study, men, obese patients, people with hypertension, smokers, and patients with low high-density lipoprotein (HDL) and high low-density lipoprotein (LDL) levels all had a higher risk of developing ACS (21). Our study showed a quite high proportion of CYP2C19^*^2/^*^3 mutant carriers. Collet et al. (22) (28%) and Amin et al. (23) (66.3%) found different proportions of CYP2C19^*^2 and CYP2C19^*^3 polymorphisms. According to a study by Sukmawan et al. (24), 11.5% of the subjects had methylation levels <50%. Almost all subjects had *CYP2C19* hypomethylation (80.1%), but only some had *P2Y12* hypomethylation (10%). In this study, the demographic, environmental, and diet factors of subjects could cause a higher proportion of *CYP2C19* hypomethylation (25). According to a study by Chen et al. (16), miRNA-26a expression was upregulated in 60.4% of subjects. Our study found that 49.8% of patients had clopidogrel resistance. Its proportion was higher in some European studies but still corresponded to clinical studies in Asia, which ranged from 20 to 65% (8).

In this study, the risk of clopidogrel resistance was lower in smokers than in non-smokers. Some studies found that the smoking habit gave an advantage known as the smoker's paradox (26, 27). A CAPRIE *post-hoc* study by Ferreiro et al. (26) found a decrease in the incidence of ischemia among smokers treated with clopidogrel compared to non-smokers (HR = 0.76, 95%CI = 0.64–0.90). *CYP2C19* and *CYP3A4* were the predominant enzymes in the biotransformation of clopidogrel. However, other isoenzymes, such as *CYP1A2* and *CYP2B6*, also contribute. Cigarette smoking is a potent inducer of *CYP1A2* (10% of CYP isoenzymes in the liver) and *CYP2B6* isoenzymes, which are involved in the first oxidative and final stages of the biotransformation of clopidogrel and increase the amount of its active metabolite (26, 27). However, it is not recommended that post-PCI patients continue smoking due to the progression of atherosclerosis, an increase in inflammatory markers, an increased risk of death, and an increased incidence of MACEs (28).

This study showed that hypomethylation of the *CYP2C19* gene body increased the risk of clopidogrel resistance. This finding corresponded with a study by Sukmawan et al. (24) that *CYP2C19* with methylation <50% had a higher risk of clopidogrel resistance than ≥50% methylated (OR = 3.1, 95%CI = 1.9–6.9, *p*-value = 0.03). DNA methylation of a gene occurs at CpG in the gene body (intragenic) or a promotor. The methylation of CpG in the promotor inhibits gene expression. There are several mechanisms by which DNA methylation can decrease gene expression. Methylation silences repetitive DNA elements, inhibiting gene transcription. Methylation can also inhibit transcription from the internal promoter. Post-transcriptional regulation can also be induced by DNA methylation, for example, alternative messenger RNA (mRNA) splicing (29). However, in the gene body, methylated CpG activates its expression. This phenomenon is known as the methylation paradox (14, 30). Previous studies revealed that the CpG island (CGI) of *CYP2C19* is located in the gene body (15, 31). The expression of *CYP2C19* will increase if it is hypermethylated, and the expression will decrease if it is hypomethylated. Reduced *CYP2C19* expression results in a decreased active metabolite of clopidogrel, which leads to clopidogrel resistance.

There was an association between miRNA-26a upregulation and clopidogrel resistance. This finding was consistent with the findings of Syam et al. (32) who showed that high miRNA-26a expression was associated with decreased inhibition of platelets by clopidogrel (OR = 4.2, *p*-value < 0.01). Chen et al. (16) found a similar result, in which platelet miRNA-26a, miRNA-199, and miRNA-23a expression was associated with clopidogrel resistance. Platelet miRNA-26a expression was associated with an increased risk of clopidogrel resistance among post-PCI patients (16). miRNAs are small non-coding RNAs that regulate gene expression by interfering with transcription or translation, thereby participating in the biological signaling pathway. miRNAs are stably present in plasma, platelets, erythrocytes, nucleated blood cells, and urine and are degraded by endogenous RNA polymerase (33). Platelet miRNA-26a expression has been shown to regulate platelet aggregation. Increased miRNA-26a expression contributes to increased VASP gene transcription. Bioinformatic analysis of the 3′-UTR region of VASP mRNA showed that miRNA-26a had a target on VASP mRNA. The western blotting results showed that the level of VASP protein and mRNA expression in platelets was significantly increased in clopidogrel resistance. VASP expression is a marker of ADP receptor activity. The active metabolite of clopidogrel blocks the ADP P2Y12 receptor, which lessens the inhibition of cyclic adenosine monophosphate- (cAMP-) dependent phosphorylation on VASP protein. High levels of VASP expression causes the protein to become more dephosphorylated, thereby triggering platelet aggregation (16).

CYP2C19^*^2/^*^3 causes the loss-of-function allele, which increases the risk of clopidogrel resistance. Clopidogrel is metabolized into active metabolites by various cytochromes in the liver, one of which is *CYP2C19*, which acts on two oxidative stages. *CYP2C19* is mainly influenced by the CYP2C19^*^2 polymorphism in exon 5, which results in protein aberrant splicing. A decrease in *CYP2C19* enzymatic activity causes a decrease in clopidogrel active metabolites, reducing the pharmacodynamic response (34). The CYP2C19^*^3 polymorphism is characterized by a point mutation in exon 4, resulting in a premature stop codon, rendering the protein formed non-functional (35). Su et al. (36) found an association between CYP2C19^*^2/^*^3 polymorphisms and an increased risk of clopidogrel resistance. The platelet aggregation method also uses a 20 μM ADP agonist, but the definition of clopidogrel resistance is different. Amin et al. (23) also showed that CYP2C19^*^2/^*^3 polymorphisms were associated with an increased risk of clopidogrel resistance when compared to the wild type.

The *P2Y12* gene encodes the ADP receptor on platelets so that the polymorphism of this gene may regulate platelet aggregation. The A57T (rs3679479) *P2Y12* polymorphism has never been associated with clopidogrel resistance. The *P2Y12* polymorphisms studied are C34T, G52T, and T744C. The A57T polymorphism is located in the intron of the *P2Y12* gene on chromosome 3, like the T744C polymorphism. Even though the location of the polymorphism is in the intron (not the part of the gene that is expressed), theory shows that the intron of a gene regulates transcription speed, chromatin modification, gene looping, mRNA stability, efficiency of mRNA translation, and regulation of splicing so that it modulates the expression of the *P2Y12* gene (37). According to a meta-analysis by Cui et al. (38), it was found that C34T and G52T polymorphisms of the *P2Y12* gene were associated with an increase in clopidogrel resistance. However, the T744C polymorphism did not give significant results. The T744C polymorphism also showed no association with clopidogrel resistance in studies in India and the USA (37, 39).

In this study, *CYP2C19* and *P2Y12* polymorphisms were not associated with clopidogrel resistance. These findings could be due to the fact that clopidogrel resistance in patients with ACS is influenced by many factors, not only by a receptor gene polymorphism but also by multiple factors. Clinical, laboratory, genetic, and epigenetic factors could affect clopidogrel resistance (40, 41). Legrand et al. (34) demonstrated a score that predicts the probability of clopidogrel resistance, called the Stent Thrombosis in Belgium (STIB) score. In the multivariate analysis, diabetes mellitus, hemoglobin <13.9 g/dl, and body mass index (BMI) > 28 kg/m^2^ were independent predictors of clopidogrel resistance. Reed et al. (42) and Nakagawa et al. (43) found that smoking might be one of the predictors of clopidogrel resistance, besides diabetes mellitus, hypertension, BMI, and renal insufficiency. According to previous studies, diabetes and smoking were important predictors of clopidogrel resistance. Insulin resistance and an increased risk of renal dysfunction among patients with diabetes mellitus could lead to an increase in platelet aggregation through *P2Y12* receptors. The characteristics of the subjects in this study may also play a role. Many subjects who are carriers of CYP2C19^*^2/^*^3 polymorphism also smoke, thus resulting in the smoker's paradox.

DNA methylation of the *P2Y12* gene was not associated with clopidogrel resistance. These results are consistent with those of Syam et al. (32), who found that the methylation of the *P2Y12* gene promoter was not associated with clopidogrel resistance in patients with ACS after primary PCI. However, Li et al. (19) found an increased risk of clopidogrel resistance in the case of the occurrence of hypomethylation in the *P2Y12* gene promoter in patients with ischemic stroke. Su et al. (44) found that a non-clopidogrel response group had lower two CpG methylation at the promoter site than a clopidogrel responsive group. The locations of CGI are different from *CYP2C19*, which is in the promoter. In contrast to the methylation of *CYP2C19* in the gene body, hypomethylation of the *P2Y12* promoter increases ADP receptor expression, decreasing the inhibition of platelet aggregation. Thus, the results of this study were allowed to differ from those of previous studies. Several factors, including demography, nutrition, and environment, influence DNA methylation (45). The use of other drugs that cause interactions, such as Calcium Channel Blocker (CCB), Proton Pump Inhibitor (PPI), Selective Serotonin Reuptake Inhibitor (SSRI), and statin, is thought to decrease clopidogrel's action in inhibiting platelet aggregation (40, 41, 46). These drugs can be competitive inhibitors of clopidogrel because they use the same CYP isoenzymes in the liver. Drug pharmacokinetic effects, such as reduced bioavailability due to absorption, are thought to influence clopidogrel resistance. Other potential genes have been shown to be associated with clopidogrel resistance. The *P2Y1* gene was not investigated in this study, but it is essential because it functions as an ADP receptor, which triggers platelet aggregation (47). GPIIb/IIIa receptor polymorphisms, which play an essential role in the later stages of platelet aggregation, may regulate clopidogrel resistance (39).

### 4.2. Association between several factors and MACEs

The exact definition of MACEs is still uncertain. However, the main conditions included in the MACE studies were angina pectoris, recurrent AMI, stroke, and death. The proportion of MACEs in previous studies showed different results (3, 4, 48). Nafrialdi et al. (3) found a proportion of 29.3% in post-PCI patients in the 3 months of observation. Miao et al. (4) and Mrdovic et al. (48) demonstrated low proportions of 1.47 and 9.1% in 4 years and 30 days of observations, respectively. Study results could differ due to different sample sizes, subject characteristics, and follow-up periods.

In this study, women marginally have a higher risk of developing MACEs than men. The proportion of women (7.5%) was smaller than that of men (92.5%), resulting in a higher risk of MACEs for women. In addition, all the women in this study had hypertension, a higher proportion of diabetes, and a higher proportion of low GFR. Age was also associated with MACEs. The severity of CHD, comorbidities, and mortality risk could increase with age (49). In laboratory parameters, there was an association between low eGFR and MACEs. A global registry study showed that renal insufficiency was an independent predictor of mortality in patients with ACS (50). Renal dysfunctions were associated with low-grade inflammation and activation of the renin-angiotensin-aldosterone system (51). In a bivariate analysis, leukocytes were not associated with MACEs in 1 year. However, in multivariate analysis, leukocytes were considered the predictors of MACEs. The leukocyte count was considered a marker of inflammation. It has been recognized that inflammation promotes the development of MACEs, especially in the initiation and progression of atherothrombosis (52).

In this study, no relation between clopidogrel resistance and MACEs is found. Several studies have linked clopidogrel resistance to MACEs based on platelet reactivity. Frere et al. (53) showed that the clopidogrel-resistant group had an 8.62 times higher risk of experiencing MACEs than the clopidogrel-sensitive group (95%CI; 2.31–32.15). Clopidogrel resistance was assessed using LTA with a 10-μM ADP agonist. Price et al. (54) also found a similar result that the clopidogrel-resistant group had a 7.17 times higher risk of experiencing MACEs (95%CI; 1.46–35.17). However, clopidogrel resistance was determined using VerifyNow *P2Y12* with a Platelet Reactivity Unit (PRU) cut-off > 235. Gurbel et al. (20) showed the association between platelet reactivity and ADP, as measured using LTA with ischemic events (MACEs) within 2 years after primary PCI. The MACE group was found to have a higher percentage of aggregation value than the non-MACE group (46 ± 14% vs. 30 ± 17%; *p* < 0.001 to ADP 5 μM and 60 ± 13% vs. 43 ± 19%, *p* < 0.001 to ADP 20 μM).

Clopidogrel resistance results in decreased inhibition of platelet aggregation, so patients have a state of high thrombogenicity, which is part of the critical pathogenesis of MACEs. Clopidogrel response is multifactorial and can be influenced by drug interactions, drug doses, adherence to clopidogrel, and genetic and epigenetic profiles, as previously described. Aghajani et al. (55) in Iran found a similar relationship between clopidogrel resistance and MACEs in patients with ACS after primary PCI who were followed up for 1 month and 3 years. Factors such as loss of follow-up in 60% of patients with clopidogrel resistance, limited patient coverage at one facility, and small sample size may influence the results of this study (55). According to this study, the number of patients resistant to clopidogrel and who experienced MACEs were low enough to potentially mask its relationship with MACEs.

According to a meta-analysis by Xi et al. (56), an increased risk of MACEs was found in the group with the loss-of-function allele of the *CYP2C19* gene. As the *CYP2C19* polymorphism is an independent factor of MACEs in patients with ACS after primary PCI, the same was studied. However, in this study, as only miRNA-26a expression and hypomethylation of *CYP2C19* were associated with clopidogrel resistance, these two factors could influence MACEs. In addition to clopidogrel resistance, several factors, such as age, comorbidities like hypertension and diabetes mellitus, smoking, blood leukocyte count, and eGFR, may impact MACEs (57). The follow-up time of 1 year could also be a factor.

The results suggested that there was an association between *CYP2C19* polymorphisms and MACEs among post-PCI patients in 1 year. According to a meta-analysis by Biswas et al. (58), an increased risk of MACEs was cumulatively shown in 12–24 months in the event of the occurrence of the *CYP2C19* polymorphism in an allele (either CYP2C19^*^2 or CYP2C19^*^3), and the risk was higher in the event of its occurrence in both alleles (OR = 2.22, 95%CI = 1.60–3.09). According to a meta-analysis by Xi et al. (56), a similar result was obtained which included Chinese, Japanese, and Korean populations. There was an increased risk of MACEs in the *CYP2C19* polymorphism group from 6 to 30 months. Collet et al. (22) found that the CYP2C19^*^2 polymorphism was associated with MACEs (death, MI, and revascularization need) within 2 years. *CYP2C19* loss-of-function polymorphism is known to be one of the clopidogrel resistance risk factors. Clopidogrel is metabolized to active metabolites by several liver cytochromes P450, one of which is *CYP2C19*, which acts in two oxidative steps (15). CYP2C19^*^2 polymorphism is located at exon 5, resulting in abnormal splicing of the enzyme, while the CYP2C19^*^3 polymorphism is located at exon 4, resulting in a premature stop codon, rendering the protein formed non-functional (34, 35). The decreased catalytic function of the enzyme results in fewer active metabolites, which reduces its capacity to inhibit platelet aggregation and increases the risk of MACEs.

However, in this study, it was found that clopidogrel resistance was not associated with MACEs. This phenomenon could be explained by the known mechanisms of the *CYP2C19* polymorphism increasing the risk of MACEs other than clopidogrel resistance. Increased inflammatory markers such as IL-6 and CRP are among them (22). Another reason is that cytochrome P450 epoxygenase is involved in the metabolism of xenobiotics. This system regulates oxidative stress, inflammation, vascular tone, hemostasis, and ischemia-reperfusion injury (59). One of the isoenzymes in this system is *CYP2C19* (60). CYP epoxygenase converts arachidonic acid into several regioisomers of epoxyeicosatrienoic acid (EET). EET has autocrine as well as paracrine effects. Endothelial EET causes vasodilation by relaxing the vascular muscles. EET is also anti-inflammatory in the vasculature and the kidneys. It stimulates angiogenesis, which protects against cardiac and brain ischemia (61). Impaired cytochrome epoxygenase enzymes are also known to promote the progression of metabolic disorders such as insulin resistance, lipid metabolism disorder, obesity, and diabetes, as well as their complications (62). If *CYP2C19* activity is reduced due to polymorphism, the protective mechanism against cardiac ischemia is also reduced. After all, MACEs are multifactorial and are influenced by clinical aspects, laboratory parameters, and genetic factors. According to previous studies, inflammation and oxidative stress are important basic mechanisms in MACEs (63–65).

The *P2Y12* polymorphism, DNA methylation of the *CYP2C19* and *P2Y12*, and miRNA-26a expression were not associated with MACEs in 1 year. These findings were different from those of several studies. Li et al. (66) found a relationship between the *P2Y12* polymorphism (C34T and G52T) and MACEs. The *P2Y12* gene polymorphism analyzed in this study was A57T, which was never linked to MACEs. Sukmawan et al. (24) found that hypomethylation of *CYP2C19* was associated with suboptimal TIMI flow after primary PCI (*p* 0.020; OR 3.4 [95%CI 1.3–8,7]). No previous studies have demonstrated the association between DNA methylation of *CYP2C19* and MACEs, such as recurrent angina, MI, stroke, or death. Li et al. (19) found the association between DNA methylation of *P2Y12* and MACEs (death, ischemic stroke, and MI). The *P2Y12* gene polymorphism and DNA methylation of *P2Y12* are related to the *P2Y12* ADP receptor, which is the target of clopidogrel, and changes in these genes can result in clopidogrel resistance. However, in this study, these two factors were not associated with clopidogrel resistance, and clopidogrel resistance was not associated with MACEs. DNA methylation of *CYP2C19* and miRNA26-26a expression were not associated with MACEs, which might be due to variable clopidogrel resistance unrelated to MACEs.

This is the first study to comprehensively evaluate clinical, laboratory, genetic, and epigenetic factors contributing to clopidogrel resistance, followed by MACEs in a 1-year observation. New findings reveal that DNA methylation of *CYP2C19* and miRNA-26a expression contribute to the development of clopidogrel resistance. Because the platelet aggregation test using LTA is quite economical, this examination is expected to be used in patient care. However, there are some limitations to this study, including the lack of attention to drug interaction and patient compliance, so further studies with a larger sample are needed to overcome these limitations. A high proportion of clopidogrel resistance in this study needs further platelet aggregation monitoring of patients. The follow-up period in this prospective cohort study may be insufficient, while some studies have lasted up to 3–4 years. Based on the high probability of developing clopidogrel resistance in the event of hypomethylation of *CYP2C19* or miRNA-26a upregulation, the authors recommend the substitution of clopidogrel for antiplatelets like ticagrelor and prasugrel.

## 5. Conclusion

One of the factors contributing to the development of MACEs among post-PCI patients with ACS is a decrease in response to clopidogrel, namely clopidogrel resistance. Genetic and epigenetic factors may regulate clopidogrel resistance. In this study, epigenetic factors such as DNA methylation of *CYP2C19* and miRNA-26a expression were associated with clopidogrel resistance. Hypomethylation of the *CYP2C19* gene body and miRNA-26a upregulation are associated with an increased risk of clopidogrel resistance. In this study, the authors found no association between clopidogrel resistance and MACEs; however, the CYP2C19^*^2/^*^3 genetic polymorphism could predict MACEs. Mutant carriers of CYP2C19^*^2/^*^3 polymorphisms are associated with an increased risk of MACEs in 1 year.

Further studies with a larger sample are required to understand genetic and epigenetic factors on clopidogrel resistance and MACEs. More research into the pathomechanisms of the *CYP2C19* polymorphism, with a focus on MACEs, is also needed. In this study, a high proportion of clopidogrel resistance requires monitoring of platelet aggregation among post-PCI patients. We also recommend the substitution of clopidogrel for ticagrelor and prasugrel in the event of hypomethylation of *CYP2C19* or miRNA-26a upregulation.

## Data availability statement

The data of this study are available from the corresponding author upon reasonable request.

## Ethics statement

The studies involving human participants were reviewed and approved by National Cardiovascular Center Harapan Kita Hospital Ethics Committee. The patients/participants provided their written informed consent to participate in this study.

## Author contributions

AG, IT, RD, and RSu conceptualized and undertook this study. RSe, IA, and AT participated in the data analysis and interpretation. AH, EL, LP, WS, and RH contributed to the composing, criticizing, and operating of the laboratory procedure, especially in genetic profiling and detection of CYP2C19 polymorphism. All authors read, revised, and approved the manuscript and ensured the integrity of all aspects of this study.

## References

[1] World Health Organization. The top 10 causes of death. Available online at: https://www.who.int/news-room/fact-sheets/detail/the-top-10-causes-of-death (accessed January 22, 2021).

[2] Levine GN Bates ER Bittl JA Brindis RG Fihn SD Fleisher LA. 2016 ACC/AHA guideline focused update on duration of dual antiplatelet therapy in coronary artery disease. JACC. (2016) 68:108–15. 10.1016/j.jacc.2016.03.513 27036918

[3] Nafrialdi N Handini NM Instiaty I Wijaya IP A. cost-effectiveness and safety analysis of dual antiplatelet therapy comparing aspirin-clopidogrel to aspirin-ticagrelor in patients with acute coronary syndrome. Med J Indones. (2018) 27:262–70. 10.13181/mji.v27i4.3024

[4] Miao B Hernandez AV Alberts MJ Mangiafico N Roman YM Coleman CI. Incidence and predictors of major adverse cardiovascular events in patients with established atherosclerotic disease or multiple risk factor. J Am Heart Assoc. (2020) 9:1014402. 10.1161/JAHA.119.014402 31937196 PMC7033849

[5] Poudel I Tejpal C Rashid H Jahan N. Major adverse cardiovascular events: an inevitable outcome of ST-elevation myocardial infarction? A literature review. Cureus. (2019) 11:e5280. 10.7759/cureus.5280 31423405 PMC6695291

[6] Zhang YJ Li MP Tang J Chen XP. Pharmacokinetic and pharmacodynamic responses to clopidogrel: evidences and perspectives. Int J Environ Res Public Health. (2017) 14:1–19. 10.3390/ijerph14030301 28335443 PMC5369137

[7] Farooq V Gogas BD Serruys PW. Restenosis: delineating the numerous causes of drug-eluting stent restenosis. Circ Cardiovasc Interv. (2011) 4:195–205. 10.1161/CIRCINTERVENTIONS.110.959882 21505166

[8] Hasan MS Basri HB Hin LP Stanslas J. Genetic polymorphisms and drug interactions leading to clopidogrel resistance: why the asian population requires special attention. Int J Neurosci. (2012) 123:143–54. 10.3109/00207454.2012.744308 23110469

[9] Sambu N Radhakrishnan A Dent H Calver AL Corbett S Gray H. Personalised antiplatelet therapy in stent thrombosis: observations from the clopidogrel resistance in stent thrombosis (CREST) registry. Heart. (2012) 98:706–11. 10.1136/heartjnl-2011-301164 22523055

[10] Rai M Gupta A McKay RG Hirst J Thompson PD Ruaño G. CYP2C19 genotype-guided antiplatelet therapy in a patient with clopidogrel resistance. Conn Med. (2012) 76:267–72. 22685981

[11] Scott S Sangkuhl K Gardner EE Stein CM Hulot JS Johnson JA. Clinical pharmacogenetics implementation consortium: clinical pharmacogenetics implementation consortium guidelines for cytochrome P450-2C19 (CYP2C19) genotype and clopidogrel therapy. Clin Pharmacol Ther. (2011) 90:328–32. 10.1038/clpt.2011.132 21716271 PMC3234301

[12] Farre AJL Tamargo J Mateos-Caceres PJ Azcona L Macaya C. Old and new molecular mechanisms associated with platelet resistance to antithrombotics. Pharm Res. (2010) 27:2365–73. 10.1007/s11095-010-0209-4 20628791

[13] Shalia KK Shah VK Pawar P Divekar SS Payannavar S. Polymorphisms of MDR1, CYP2C19 and P2Y12 genes in indian population: effects on clopidogrel response. Indian Heart J. (2013) 65:158–67. 10.1016/j.ihj.2013.02.012 23647895 PMC3861302

[14] Moosavi A Ardekani AM. Role of epigenetics in biology and human diseases. Iran Biomed J. (2016) 20:246–58. 27377127 10.22045/ibj.2016.01 PMC5075137

[15] Burns KE Shepherd P Finlay G Tingle MD Helsby NA. Indirect regulation of *CYP2C19* gene expression via DNA methylation. Xenobiotica. (2017) 1:1–12. 10.1080/00498254.2017.1372648 28840784

[16] Chen S Qi X Chen H Li M Gu J Liu C. Expression of miRNA-26a in platelets is associated with clopidogrel resistance following coronary stenting. Exp Ther Med. (2016) 12:518–24. 10.3892/etm.2016.3278 27347088 PMC4907078

[17] Neki NS. Clopidogrel resistance: current Issues. J Enam Med Coll. (2016) 6:38. 10.3329/jemc.v6i1.26381

[18] Zoheir N Elhamid SA Abulata N Sobky ME Khafagy D Mostafa A. P2Y12 receptor gene polymorphism and antiplatelet effect of clopidogrel in patients with coronary artery disease after coronary stenting. Blood Coagul Fibrinolysis. (2013) 24:525–31. 10.1097/MBC.0b013e32835e98bf 23751603

[19] Li XG Ma N Wang B Li XQ Mei SH Zhao K. The impact of P2Y12 promoter DNA methylation on the recurrence of ischemic events in chinese patients with ischemic cerebrovascular disease. Sci Rep. (2016) 6:34570. 10.1038/srep34570 27686864 PMC5043343

[20] Gurbel PA Antonio MJ Bliden KP Dichiara J Suarez TA Singla A. Platelet reactivity to adenosine diphosphate and long-term ischemic event occurence following percutaneous coronary intervention: a potential antiplatelet therapeutic target. Platelets. (2008) 19:595–604. 10.1080/09537100802351065 19012177

[21] Hubacek JA Stanek C Gebauerova M Adamkova V Lesauskaite V Peksiene DZ. Traditional risk factors of acute coronary syndrome in four different male populations–total cholesterol value does not seem to be relevant risk factor. Physiol Res. (2017) 66:S121–8. 10.33549/physiolres.933597 28379037

[22] Collet JP Hulot JS Pena A Villard E Esteve JB Silvain J. Cytochrome P450 2C19 polymorphism in young patients treated with clopidogrel after myocardial infarction: a cohort study. Lancet. (2009) 373:309–17. 10.1016/S0140-6736(08)61845-0 19108880

[23] Amin AA Chin LS Noor DAM Mostada H Kader MASKA Hay YK. The effect of CYP2C19genetic polymorphism and non-genetic factors on clopidogrel platelets inhibition in East Asian coronary artery disease patients. Thrombosis Res. (2017) 158:22–4. 10.1016/j.thromres.2017.07.032 28802144

[24] Sukmawan R Hoetama E Danny SS Giantini A Listianingsih E Rejeki VG. Increase in the risk of clopidogrel resistance and consequent TIMI flow impairment by DNA hypomethylation of CYP2C19 gene in STEMI patients undergoing primary percutaneous coronary intervention (PPCI). Pharmacol Res Perspect. (2021) 9:e00738 10.1002/prp2.738 33641235 PMC7915409

[25] Lim U Song MA. Dietary and lifestyle factors of DNA methylation. Methods Mol Biol. (2012) 83:357–76. 10.1007/978-1-61779-612-8_23 22359306

[26] Ferreiro JL Bhatt DL Ueno M Bauer D Angiolillo DJ. Impact of smoking on long-term outcomes in patients with Atherosclerotic vascular disease treated with aspirin or Clopidogrel. JACC. (2014) 63:769–77. 10.1016/j.jacc.2013.10.043 24239662

[27] Edem E Kirdok AH Kinay AO Tekin UI. Does “smoker's paradox” exist in clopidogrel-treated Turkish patients with acute coronary syndrome. Platelets. (2015) 27:1–5. 10.3109/09537104.2015.1083544 26367336

[28] Nakanishi R Berman DS Budoff MJ Gransar H Achenbach S Al-Mallah M. Current but not past smoking increases the risk of cardiac events: insights from coronary computed tomographic angiography. Eur Heart J. (2015) 36:1031–40. 10.1093/eurheartj/ehv013 25666322 PMC4416139

[29] Shenker N Flanagan JM. Intragenic DNA methylation: implications of this epigenetic mechanism for cancer research. Br J Cancer. (2012) 106:248–53. 10.1038/bjc.2011.550 22166804 PMC3261681

[30] Bibikova M Barnes B Tsan C Ho V Klotzle B Le JM. High density DNA methylation array with single CpG site resolution. Genomics. (2011) 98:288–95. 10.1016/j.ygeno.2011.07.007 21839163

[31] Tang X Ge L Chen Z. Methylation of the constitutive androstane receptor is involved in the suppression of CYP2C19 in hepatitis B virus-associated hepatocellular carcinoma. Drug Metab Dispos. (2016) 44:1643–52. 10.1124/dmd.116.070243 27440862

[32] Syam H Sukmawan R Dharma S Alaztha G Giyantini A Prakoso R. Epigenetic interaction of miRNA-26a and P2Y12 gene DNA methylation on platelet reactivity under clipodiogrel and their impact to coronary flow after primary PCI in STEMI. Eur Heart J. (2020) 41:ehaa946.1547. 10.1093/ehjci/ehaa946.1547

[33] Berezikov E Cuppen E Plasterk RHA. Approaches to microRNA discovery. Nat Genet. (2006) 38:S2–7. 10.1038/ng1794 16736019

[34] Legrand D Barbato E Chenu P Magne J Vrolix M Wijns W. The STIB score: a simple clinical test to predict clopidogrel resistance. Acta Cardiol. (2015) 70:516–21. 10.1080/AC.70.5.3110511 26567810

[35] Dehbozorgi M Kamalidehghan B Hosseini I Dehghanfard Z Sangtarash MH Firoozi M. Prevalence of the CYP2C19^*^2 (681 G>A), ^*^3 (636 G>A) and ^*^17 (-806 C>T) alleles among an Iranian population of different ethnicities. Mol Med Rep. (2018) 17:41955–202. 10.3892/mmr.2018.8377 29328413 PMC5802190

[36] Su Q Li J Tang Z Yang S Xing G Liu T. Association of CYP2C19 polymorphism with clopidogrel resistance in patients with acute coronary syndrome in china. Med Sci Monit. (2019) 25:7138–48. 10.12659/MSM.915971 31543510 PMC6775793

[37] Shaul O. How introns enhance gene expression. Int J Biochem Cell Biol. (2017) 91:145–55. 10.1016/j.biocel.2017.06.016 28673892

[38] Cui G Zhang S Zou J Chen Y Chen H. P2Y12 receptor gene polymorphism and the risk of resistance to clopidogrel: A meta-analysis and review of the literature. Adv Clin Exp Med. (2017) 26:343–49. 10.17219/acem/63745 28791856

[39] Angiolillo DJ Fernandez-Ortiz A Bernardo E Ramirez C Cavallari U Trabetti E. Lack of association between the P2Y12 receptor gene polymorphism and platelet response to clopidogrel in patients with coronary artery disease. Thromb Res. (2005) 116:491–7. 10.1016/j.thromres.2005.03.001 16181985

[40] Cardoso RN Benjo AM DiNicolantonio JJ Garcia DC Macedo FYB El-Hayek G. Incidence of cardiovascular events and gastrointestinal bleeding in patients receiving clopidogrel with and without proton pump inhibitors: an updated meta-analysis. Open Heart. (2015) 2:e000248. 10.1136/openhrt-2015-000248 26196021 PMC4488889

[41] Feher G Feher A Pusch G Koltai K Tibold A Gasztonyi B. Clinical importance of aspirin and clopidogrel resistance. World J Cardiol. (2010) 2:171–86. 10.4330/wjc.v2.i7.171 21160749 PMC2998916

[42] Reed GW Cannon CP Waalen J Teirstein PS Tanguay JF Berger PB. Influence of smoking on the antiplatelet effect of clopidogrel differs according to clopidogrel dose:insights from the GRAVITAS Trial. Catheter Cardiovasc Interv. (2017) 89:190–8. 10.1002/ccd.26428 26909669

[43] Nakagawa I Park HS Yokoyama S Wada T Hironaka Y Motoyama Y. Influence of diabetes mellitus and cigarette smoking on variability of the clopidogrel-induced antiplatelet effect and efficacy of active management of the target P2Y12 reaction unit range in patients undergoing neurointerventional procedures. J Stroke Cerebrovasc Dis. (2016) 25:16–71. 10.1016/j.jstrokecerebrovasdis.2015.09.010 26493334

[44] Su J Li X Yu Q Liu Y Wang Y Song H. Association of P2Y12 gene promoter DNA methylation with the risk of clopidogrel resistance in coronary artery disease patients. BioMed Res Int. (2014) 2014:1–8. 10.1155/2014/450814 24745016 PMC3976931

[45] El-Maarri O Becker T Junen J Manzoor SS Diaz-Lacava A Schwaab R. Gender specific differences in levels of DNA methylation at selected loci from human total blood: a tendency toward higher methylation levels in males. Hum Genet. (2007) 122:505–14. 10.1007/s00439-007-0430-3 17851693

[46] Pedersen F Butrymovich V Kelbaek H Wachtell K Helqvist S Katrup J. Short and long therm cause of death in patients treated with primary PCI for STEMI. JACC. (2014) 64:2101–8. 10.1016/j.jacc.2014.08.037 25457398

[47] Kar R Meena A Yadav BK Yadav R Kar SS Saxena R. Clopidogrel resistance in North Indian patients of coronary artery disease and lack of its association with platelet ADP receptors P2Y1 and P2Y12 gene polymorphisms. Platelets. (2012) 24:297–302. 10.3109/09537104.2012.693992 22721490

[48] Mrdovic I Savic L Krljanac G Asamin M Perunicic J Lasica R. Predicting 30-day major adverse cardiovascular events after primary percutaneous coronary intervention the RISK-PCI score. Int J Cardiol. (2013) 162:220–7. 10.1016/j.ijcard.2011.05.071 21663982

[49] Nakazato R Arsanjani R Achenbach S Gransar H Cheng VY Dunning A. Age-related risk ofmajor adverse cardiac event risk and coronary artery disease extent and severity by coronary CT angiography: results from 15 187 patients from the International Multisite CONFIRM Study. Eur Heart J. (2014) 15:586–94. 10.1093/ehjci/jet132 24714312 PMC3979454

[50] Santopinto JJ Fox KA Goldberg RJ Budaj A Piñero G Avezum A. Creatinine clearance and adverse hospital outcomes in patients with acute coronary syndromes: findings from the global registry of acute coronary events (GRACE). Heart. (2003) 89:1003–8. 10.1136/heart.89.9.1003 12923009 PMC1767853

[51] Anavekar NS Solomon SD McMurray JJ Maggioni A Rouleau JL Califf R. Comparison of renal function and cardiovascular risk following acute myocardial infarction in patients with and without diabetes melitus. Am J Cardiol. (2008) 101:925–9. 10.1016/j.amjcard.2007.11.037 18359309

[52] Libby P. Inflammation in atherosclerosis. Nature. (2002) 420:868–74. 10.1038/nature01323 12490960

[53] Frere C Cuisset T Quilici J. ADP-induced platelet aggregation and platelet reactivity index VASP are good predictive markers for clinical outcomes in nonST elevation acute coronary syndrome. Thromb Haemost. (2007) 98:838–43. 10.1160/TH07-04-0296 17938809

[54] Price MJ Endemamm S Gollapudi RR. Prognostic significance of postclopidogrel platelet reactivity assessed by a point-of-care assay on thrombotic events after drug-eluting stent implantation. Eur Heart J. (2008) 29:992–1000. 10.1093/eurheartj/ehn046 18263931

[55] Aghajani MH Kobarfard F Shojaei SP Ahmadour F Safi O Kazemina N. The impact of clopidogrel resistance on clinical outcome of iranian patients undergoing percutaneous coronary intervention. Iran J Pharmacol Res. (2018) 17:1099–104. 30127832 PMC6094421

[56] Xi Z Fang F Wang J AlHelal J Zhou Y Liu W. CYP2C19 genotype and adverse cardiovascular outcomes after stent implantation in clopidogrel-treated Asian populations: A systematic review and meta-analysis. Platelets. (2019) 30:229–40. 10.1080/09537104.2017.1413178 29257922

[57] Huang JC Kuo IC Tsai YC Lee JJ Lim LM Chen SC. Variability Predicts Major Adverse Cardiovascular Events and Hospitalization in Maintenance Hemodialysis Patients. Kidney Blood Press Res. (2017) 42:76–88. 10.1159/000469716 28315879

[58] Biswas M Kali SK. Association of CYP2C19 loss-of-function alleles with major adverse cardiovascular events of clopidogrel in stable coronary artery disease patients undergoing percutaneous coronary intervention: meta-analysis. Cardiovasc Drugs Ther. (2021). 10.1007/s10557-021-07142-w 33523336

[59] Jeong YH Tantry US Kim IS Koh JS Kwon TJ Park Y. Effect of CYP2C19^*^2 and ^*^3 loss-of-function alleles on platelet reactivity and adverse clinical events in east asian acute myocardial infarction survivors treated with clopidogrel and aspirin. Circ Cardiovasc Interv. (2011) 4:585–94. 10.1161/CIRCINTERVENTIONS.111.962555 22045970

[60] Shahabi P Siest G Meyer UA Visvikis-Siest S. Human cytochrome P450 epoxygenases: Variability in expression and role in inflammation-related disorders. Pharmacol Ther. (2014) 144:134–61. 10.1016/j.pharmthera.2014.05.011 24882266

[61] Spector AA Kim HY. Cytochrome P450 epoxygenase pathway of polyunsaturated fatty acid metabolism. Biochim Biophys Acta. (2015) 1851:356–65. 10.1016/j.bbalip.2014.07.020 25093613 PMC4314516

[62] Xu X Li R Hoopes SL Zeldin DC Wang DW. The role of cytochrome P450 epoxygenases, soluble epoxide hydrolase, and epoxyeicosatrienoic acids in metabolic diseases. Adv Nutr. (2016) 7:1122–8. 10.3945/an.116.012245 28140329 PMC5105036

[63] Dharma S Hapsari R Siswanto BB Laarse A. Blood leukocyte count on admission predicts cardiovascular events in patients with acute non-ST elevation myocardial infarction. Int J Angiol. (2015) 24:127–32. 10.1055/s-0035-1544178 26060384 PMC4452600

[64] Mohammad AM Al-Allawi NAS. CYP2C19 genotype is an independent predictor of adverse cardiovascular outcome in Iraqi patients on clopidogrel post percutaneous coronary intervention. J Cardiovasc Pharmacol. (2017) 71:347–51. 10.1097/FJC.0000000000000577 29554005

[65] Tsai IT Wang CP Lu YC Hung WC Wu CC Lu LF. The burden of major adverse cardiac events in patients with coronary artery disease. BMC Cardiovasc Disord. (2017) 17:1–13. 10.1186/s12872-016-0436-7 28052754 PMC5210314

[66] Li M Wang H Xuan L Shi X Zhou T Zhang N. Associations between P2RY12 gene polymorphisms and risks of clopidogrel resistance and adverse cardiovascular events after PCI in patients with acute coronary syndrome. Medicine (Baltimore). (2017) 96:1–6. 10.1097/MD.0000000000006553 28383427 PMC5411211

